# Host gene expression in the Nasopharynx can discriminate microbiologically confirmed viral and bacterial lower respiratory tract infection

**DOI:** 10.1017/cts.2025.10191

**Published:** 2025-10-29

**Authors:** L. Gayani Tillekeratne, Nicholas O’Grady, Maria D. Iglesias-Ussel, Jack Anderson, Alana Brown, Armstrong Obale, Christina Nix, Champica K. Bodinayake, Ajith Nagahawatte, Robert Rolfe, E. Wilbur Woodhouse, Gaya B. Wijayaratne, Senali Weerasinghe, U.H.B.Y. Dilshan, Jayani Gamage, Ruvini Kurukulasooriya, Madureka Premamali, Himali S. Jayasinghearachchi, Bradly P. Nicholson, Emily R. Ko, Ephraim L. Tsalik, Micah T. McClain, Rachel A. Myers, Christopher W. Woods, Thomas W. Burke

**Affiliations:** 1 Duke University School of Medicine, Durham, NC, USA; 2 Duke Global Health Institute, Durham, NC, USA; 3 Faculty of Medicine, University of Ruhuna, Karapitiya, Galle, Sri Lanka; 4 Duke-Ruhuna Collaborative Research Centre, Faculty of Medicine, University of Ruhuna, Karapitiya, Galle, Sri Lanka; 5 Faculty of Medicine, General Sir John Kotelawala Defence University, Ratmalana, Sri Lanka; 6 Institute for Medical Research, Durham Veterans Affairs Medical Center, Durham, NC, USA; 7 Danaher Corporation, Washington, DC, USA

**Keywords:** Lower respiratory tract infection, host response, antimicrobial stewardship, nasopharynx, rapid diagnostic

## Abstract

**Introduction:**

Distinguishing viral versus bacterial lower respiratory tract infection (LRTI) is challenging. We previously developed a rapid, host response-based test (Biomeme HR-B/V assay) using peripheral blood samples to identify viral versus bacterial infection. We assessed the performance of this assay when using nasopharyngeal (NP) samples.

**Methods:**

Patients with LRTI were enrolled, and a NP swab sample was run using the HR-B/V assay (assessing 24 gene targets) on the Franklin^TM^ platform. The performance of the prior classifier at identifying viral versus bacterial infection was assessed. A novel predictive model was generated for NP samples using the same 24 targets. Results were validated using external datasets with nasal/NP RNA sequence data.

**Results:**

Nineteen patients (median age 62 years, 52.1% male) were included. When using the prior HR-B/V classifier on NP samples of 19 patients with LRTI (12 viral, 7 bacterial), the area under the receiver operator curve (AUC) for viral versus bacterial infection was 0.786 (0.524–1), with accuracy 0.79 (95% CI 0.57–0.91), positive percent agreement (PPA) 0.43 (95% CI 0.16–0.75), and negative percent agreement (NPA) 1.00 (95% CI 0.76–1). The novel model had AUC 0.881 (95% CI 0.726–1), accuracy 0.84 (95% CI 0.62–0.94), PPA 0.86 (95% CI 0.49–0.97), and NPA 0.83 (95% CI 0.55–0.95) for bacterial infection. Validation in two external datasets showed AUC of 0.932 (95% CI 0.90–0.96) and 0.915 (95% CI 0.88–0.95).

**Conclusions:**

We show that host response in the nasopharynx can distinguish viral versus bacterial LRTI. These findings need to be replicated in larger cohorts with diverse LRTI etiologies.

## Introduction

Identifying the etiology of lower respiratory tract infection (LRTI), which includes syndromes such as bronchitis, pneumonia, and infectious exacerbations of asthma and chronic obstructive pulmonary disease (COPD), remains challenging. Viral and bacterial LRTI present with similar clinical signs and symptoms, leading clinicians to prescribe antibacterials for fear of missing an otherwise fatal bacterial infection [1].

LRTI diagnostics that are currently used in clinical care, such as sputum or blood cultures or multiplex polymerase chain reaction (PCR) of nasopharyngeal (NP) or sputum samples, are generally focused on identifying a specific viral or bacterial pathogen. However, such pathogen-based diagnostics can have low sensitivity (in the case of culture), may detect only a select set of pathogens (in the case of PCR), or fail to distinguish colonization from infection (in both cases) [2,3]. Moreover, identifying an organism from a non-invasive, upper respiratory sample such as nasal or NP sample may not reflect the etiology of infection in the lower respiratory tract [4]. Host response-based diagnostics, which assess the host’s response to infection and broadly classify infection as viral or bacterial, provides important adjunctive information to pathogen-based diagnostics, and can also help differentiate colonization from infection. However, traditional host response-based diagnostics, which include protein biomarkers such as C-reactive protein (CRP) and procalcitonin (PCT), have also been plagued by poor performance characteristics [5]. Newer response-based tests that assess multiple protein biomarkers, such as the FebriDx (Lumos Diagnostics) and the MeMed BV (MeMed) tests, may have improved performance characteristics, but are not yet widely used in clinical practice [6,7].

In recent years, measuring host gene expression has emerged as a novel strategy for assessing host response [8]. Transcriptomics-based tests may have superior performance characteristics to traditional protein-based host response tests. One such transcriptomics test, the TriVerity by Inflammatix, was recently cleared by the US Food and Drug Administration (FDA) for distinguishing acute viral from bacterial infection [9].

We have previously developed a blood-based gene expression classifier (Biomeme HR-B/V test) using 22 gene targets and 2 normalizing genes that is run on a rapid, real-time quantitative polymerase chain reaction (RT-qPCR) platform, the Biomeme Franklin^TM^ [10]. The accuracy of the Biomeme HR-B/V test at distinguishing viral versus bacterial infection was 85%. For acute respiratory infections, assessing host response in the nasopharynx is an appealing strategy, as measuring localized host response may allow earlier detection of infection. In addition, a NP sample affords the possibility of developing an integrated diagnostic that can assess both pathogen and host response using a single, non-invasively collected sample [11]. Others have shown that gene expression classifiers using nasal or NP samples can differentiate viral respiratory infection from non-viral respiratory infection, healthy controls, or between different types of viral respiratory infection [11–14]. The performance of nasal or NP-based gene expression classifiers at distinguishing viral versus bacterial respiratory infection is just starting to be explored [15].

In this study, we enrolled patients with LRTI and assessed the performance of our previously developed blood-based classifier at identifying viral versus bacterial infection when applied to NP samples.

## Methods

### Subject recruitment

Consecutive patients ≥ 1 year old admitted with acute LRTI to an 1800-bed, public tertiary care hospital in Southern Province, Sri Lanka were identified for enrollment within 48 hours of admission during the period of November 2019 to July 2020. Patients were eligible if they met an age-specific case definition for LRTI and had an acute illness, as described previously [16]. Chest X-ray imaging within 48 hours of admission was required for eligibility for patients ≥ 5 years, but was not required in patients < 5 years of age. Patients were not eligible to participate in this study if they were outpatients, hospitalized within the past 28 days, or had known or suspected infections at other anatomic sites requiring antibacterial therapy. Written informed consent was obtained from all patients or their guardians for children < 18 years of age. Written assent was obtained from children 12–17 years of age.

### Collection of clinical information and biological samples

At enrollment, a standardized questionnaire was administered by trained research assistants to collect demographic and clinical information. Laboratory tests results obtained during hospitalization as part of routine clinical care, such as white blood cell count and CRP level, were also recorded. Two NP swab samples were obtained, with one placed in universal transport media (UTM) and the other placed in RNAlater^®^ RNA stabilization solution (Thermo Fisher Scientific). A urine sample was also collected. All samples were stored at -80°C until used for testing. All patients also had blood and sputum samples collected for culture at enrollment, and these were processed immediately.

### Etiological testing

The NP sample stored in UTM was tested for 3 bacterial and 18 viral pathogens using the Luminex NxTAG Respiratory Pathogen Panel (Luminex Corporation, Austin, TX, USA). Testing for SARS-Coronavirus-2 was conducted using the Centers for Disease Control and Prevention (CDC) SARS-CoV-2 assay on an AB7500 Fast DX (Applied Biosystems, Waltham, MA, USA). Urine antigen testing for *Streptococcus pneumoniae* was performed using the BinaxNOW (Abbott, Chicago, IL, USA). Sputum and blood cultures were processed manually using standard microbiological techniques according to the Clinical Laboratory Standards Institute [17,18].

### Clinical adjudications

Clinical adjudication served as the comparator method to determine the etiology of illness. Adjudicators were physicians with experience in the diagnosis of infectious diseases, and used a combination of clinical history, results from laboratory and radiographic tests performed for clinical care, and results from etiological tests performed for research purposes to conduct adjudications. Two adjudicators independently determined the likelihood of bacterial and/or viral infection, non-infectious syndrome, or indeterminate diagnosis. Adjudicator discordance was resolved by a consensus panel of at least three experts, with simple majority determining final diagnosis [19,20]. Among those who had a bacterial or viral infection identified as the primary cause, microbiological level of confidence was identified as being high confidence (positive microbiological data with supportive clinical history) versus low confidence (negative microbiological data but with supportive clinical history).

### Selection of subjects into sub-analysis

Subjects were selected for inclusion into this sub-analysis if they had a bacterial or viral infection identified as the primary cause of infection, and if the microbiological level of confidence was identified as being high (positive microbiological data with supportive clinical history). A selection of 32 patients who met these criteria were initially selected at random for testing, based on sample and resource availability.

### Platform and classifier for assessing host gene expression

The biomeme Franklin^TM^ molecular diagnostic platform can detect up to 27 targets per sample through RT-qPCR. The biomeme HR-B/V test on this platform includes 22 discriminating targets (BATF, CFAP45, CTBP1, DEFA3, DSC2, EXOG, FOLR3, GCAT, HLA-DRB1, IFI27, LAMP1, LAPTM4B, MCTP1, OAS3, PLAC8, RPS21, SIGLEC1, SIRPB1, SLC29A1, STAP1, TNFAIP2, USP18) along with two normalization controls (DECR1 and PPIB) and an RNA process control (RNA extraction and RT-PCR control utilizing MS2 bacteriophage) [10].

### RNA extraction and RT-qPCR

Banked NP samples collected in RNAlater^®^ were thawed with 250–500µl of solution warmed at 37°C to return the precipitated reagent into solution. This was then centrifuged for 5 minutes at 3000 × G to pellet nucleic acid-containing material. The RNAlater^®^ supernatant was aspirated and the pellet was resuspended in 500µl of Biomeme Lysis Buffer (Biomeme, Philadelphia, USA). The whole volume was then added to an M1 RNA 2.0 Sample Prep Cartridge (Biomeme, Philadelphia, USA) for extraction and was eluted into 400µL TE buffer. Sample was pumped through the Biomeme M1 sample prep column, which contains silica membranes, a barbed tip, and Luer lock for attachment to a 1 mL syringe. The column’s barbed tip pierces the foil sealed cartridge chambers, which contain lysis buffer, protein, salt wash and drying buffers. For the final air-drying step, we transferred the column to a clean 20 mL syringe and dried it onto a clean low lint wipe with 5–10 pumps, and eluted the RNA with 400µL 10 mM Tris-HCl, 0.1 mM EDTA buffer. Purified RNA samples were added to lyophilized HR-B/V assay reagents, then run on the Franklin^TM^ three9 thermocycler (Biomeme, Philadelphia, USA). Primers/probes were multiplexed for triplex reactions.

### Statistical analysis

#### Data processing

Raw relative fluorescent units (RFU) were exported from the Biomeme Franklin™ mobile RT-qPCR thermocyclers to a cloud database. Values were converted to cycle threshold (Ct) units and exported via XML worksheets. Samples that had greater than 33% of their target Ct values missing were removed from downstream analysis. After sample removal, gene targets with missing Ct values in 33% of all samples were removed for new model developments. However, all targets were considered when using the existing blood-derived models that are used with the Biomeme Franklin^TM^. These existing models include separate models for bacterial versus nonbacterial and viral versus non-viral infections [10]. Missing or non-detected values were imputed to the maximum observed value per target plus one cycle threshold, *i.e.*, max (observed Ct) + 1. RT-PCR values were normalized with the delta Ct method, which is the target Ct value minus the mean of the reference targets (DECR1 and PPIB) for that sample [21].

#### Exploratory and differential expression analysis

Principal component analysis (PCA) plots were generated for dimensionality reduction, separated by preservation type, and further stratified by their clinical bacterial and viral adjudications. Differential expression between bacterial and viral samples for each target was assessed using a two sample *t*-test. *P*-values were adjusted for multiple testing using the Benjamini-Hochberg procedure and targets with adjusted *p*-value ≤ 0.05 were considered significant [22].

#### Pathway enrichment analysis

Over-representation analysis was performed comparing these 22 genes from the Biomeme HR-B/V assay to a universe of all transcripts from the org. Hs.eg.db R object [23]. All genes were passed into clusterProfiler and run against the Gene Ontology biological processes (BP) database [24]. Enrichment results were limited to pathways that had three or more target genes in a pathway, and additionally restricted to a false discovery corrected *p*-value ≤ 0.05.

#### Predictive modeling using existing blood-based Biomeme HR-B/V Franklin^TM^ models

The HR-BV blood-derived models included two previously developed models for bacterial versus non-bacterial and viral versus non-viral infections. The bacterial model assesses bacterial infection versus non-bacterial infection, and the viral model assesses viral infection versus non-viral infection. Including two distinct models allows for the possibility of assessing bacterial and viral co-infection. Methods for deriving these models are published elsewhere [10]. These sparse logistic regression models were used in predictions on the normalized NP sample data. Box plots and area under the receiver operator curves (AUC) were built to assess performance.

#### Retraining a predictive model of viral versus bacterial infection

In addition to the imported Biomeme HR-B/V Franklin^TM^ models, a new model was built using linear sparse logistic regression on NP data. Specifically, bacterial versus viral elastic net regularization model favoring ridge regression (*α* = 0.1) was implemented in the glmnet R package [25]. The optimal regularization parameter (λ) was obtained via leave-one-out cross-validation (LOOCV). Estimated performance metrics included AUC, accuracy, positive percent agreement (PPA), negative percent agreement (NPA), and box plots of predicted probabilities for bacterial versus viral infection. The threshold for bacterial classifiers for determining accuracy, PPA, and NPA was estimated via the Youden Index [26]. Confidence intervals were generated from confusion matrices using epiR, Wilson method [27]. Gene-specific model weights were averaged over all iterations. All statistical analyses were completed using R Statistical Software version 4.4.1 [28].

#### Performance of standard biomarker - CRP

The performance characteristics of the commonly used biomarker CRP at identifying viral versus bacterial infection in our cohort were determined using CRP test results that were obtained during routine clinical care. AUC, PPA, NPA, positive predictive value (PPV), and negative predictive value (NPV) were determined for CRP. The performance of the novel viral versus bacterial model was compared with that of CRP using the DeLong test to compare AUCs, and a test of two proportions for PPA, NPA, PPV and NPV.

#### External validation

Series matrix gene counts and associated phenotypic data from series GSE163151 and GSE188678 were downloaded from the Gene Expression Omnibus (GEO) database. Data was converted into EdgeR objects for pre-processing quality control [29]. Lowly expressed genes were filtered using EdgeR’s filterByExpr function, and expression counts were normalized using the trimmed mean of M-values method (TMM) [30]. Density plots across raw, filtered, and log2-counts per million (cpm) normalized data, and PCA on log2-cpm data were generated for study design considerations and removal of outliers. Voom weights were estimated to control for mean-variance heteroscedasticity, and incorporated in subsequent differential expression analysis [31]. Log2 normalized transcriptomic data was then filtered down to the 22 genes present in the Biomeme HR-B/V assay.

Elastic net logistic regression models for each GEO set were built using the 22 Biomeme HR-B/V genes, excluding housekeeping genes DECR1 and PPIB, after quality control filtering. The models were set up to predict viral versus non-viral samples and built using the process described above. We consider AUC and probabilities of viral infection summarized as box plots. Model coefficients (target weights) for all iterations were used to summarize (as boxplots and LOOCV usage) the targets used by the model.

### Ethical considerations

This study was approved by the Ethical Review Committee of the Faculty of Medicine, University of Ruhuna, Sri Lanka (application number 15.02.2018.3.13) and the Duke University Institutional Review Board (Pro00092502) and conducted in accordance with the principles outlined in the Declaration of Helsinki. All participants provided written informed consent prior to participating in the study.

## Results

### Expression of gene targets

A subset of 32 subjects who had an etiology of viral (23) or bacterial (9) infection with high level of microbiological confidence based on clinical adjudications was initially identified. A total of 13 NP samples (11 viral and 2 bacterial, 41% of total samples) had > 33% of their targets missing by the HR-B/V test and were excluded from subsequent analyses (Supplementary Figure 1). The analysis cohort thus included 19 subjects (12 viral and 7 bacterial infections), with specimens collected only at initial enrollment. The sociodemographic and clinical characteristics of the 19 subjects are shown in Table 1.

**Table 1.** Sociodemographic and clinical characteristics of subjects with viral or bacterial etiology of lower respiratory tract infection based on clinical adjudications. The frequency (percentage) or median (interquartile range) is displayed

	Etiology of infection based on clinical adjudication		
Characteristic	Overall, *n* = 19	Viral, *n* = 12	Bacterial, *n* = 7
Age (years)	61 (31–66)	41 (25–64)	66 (63–69)
Male sex	8 (52.1%)	5 (41.7%)	3 (42.9%)
Sinhalese ethnicity	19 (100.0%)	12 (100.0%)	7 (100.0%)
History of chronic diseases Asthma/ COPD Ischemic heart disease/ HTN/ CHF Diabetes mellitus Immunosuppression in past 30 days Cancer	11 (57.9%) 8 (42.1%) 4 (21.1%) 0 (0.0%) 1 (5.3%)	7 (58.3%) 5 (41.7%) 1 (8.3%) 0 (0.0%) 1 (8.3%)	4 (57.1%) 3 (42.9%) 3 (42.9%) 0 (0.0%) 0 (0.0%)
Days of illness at enrollment	7 (5–16)	7 (5–11)	14 (6–17)
Fever during illness	16 (84.2%)	11 (91.7%)	5 (71.4%)
Antibiotic use prior to admission (yes or unsure)	16 (84.2%)	10 (83.3%)	6 (85.7%)
Admission clinical diagnosis Lower respiratory tract infection Upper respiratory tract infection COPD exacerbation Asthma exacerbation Unspecified viral fever* Dengue Other	12 (63.2%) 1 (5.3%) 1 (5.3%) 3 (15.8%) 1 (5.3%) 1 (5.3%) 1 (5.3%)	8 (66.7%) 1 (8.3%) 1 (8.3%) 1 (8.3%) 1 (8.3%) 1 (8.3%) 0 (0.0%)	4 (57.1%) 0 (0.0%) 0 (0.0%) 2 (28.6%) 0 (0.0%) 0 (0.0%) 1 (14.3%)
Microbiological etiology of illness (confirmed by clinical adjudications) Adenovirus Human enterovirus/ rhinovirus Influenza A/B Parainfluenza 1–4 *Mycoplasma pneumoniae* *Staphylococcus aureus* *Streptococcus pneumoniae*	— — — — — — —	1 (8.3%) 2 (16.7%) 6 (50.0%) 3 (25.0%) — — —	— — — — 1 (14.3%) 1 (14.3%) 5 (71.4%)

Abbreviations: COPD = chronic obstructive pulmonary disease; HTN = hypertension; CHF = congestive heart failure.

* One participant had an admission clinical diagnosis of dengue versus an unspecified viral fever (both diagnoses were listed).

**Figure 1.**
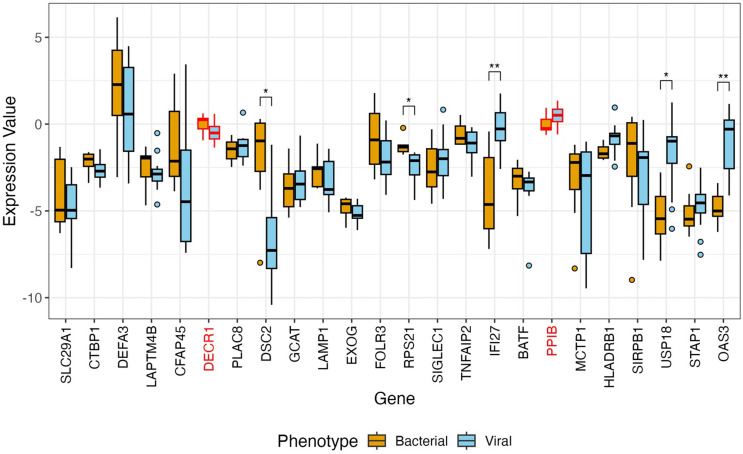
Normalized expression of genes in nasopharyngeal samples in subjects with lower respiratory tract infection, differentiated by viral (*n* = 12) versus bacterial (*n* = 7) infection. Expression values are qPCR cycle thresholds multiplied by negative one. The genes listed in red are the normalizing genes. Genes denoted with a single asterisk have a differentially expressed adjusted *p*-value of ≤ 0.05, while genes denoted with a double asterisk have an adjusted *p*-value ≤ 0.01.

When comparing gene expression in viral versus bacterial infection, five classifier genes (OAS3, IFI27, USP18, DSC2, RSP21) were significantly differentially expressed (Figure 1). The three most differentially expressed genes (OAS3, IFI27, and USP18) were viral targets and showed higher expression in viral samples in comparison to bacterial samples. The remaining classifier genes and two normalizing genes (DECR1 and PPIB) were not significantly expressed, all with adjusted *p*-values ≥ 0.25.

### Pathway analysis

**Figure 2.**
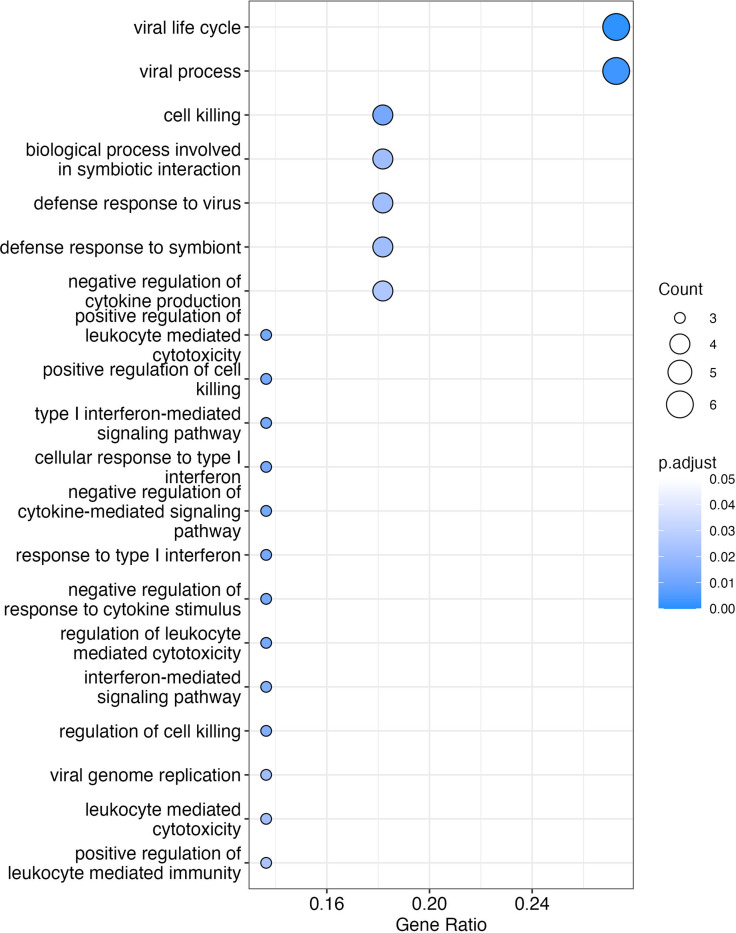
Pathways in which the 22 genes represented in the Biomeme HR-B/V classifier were found at a statistically significant level compared to other pathways. The 20 pathways with highest statistical significance are displayed here.

Of the pre-selected 22 genes from the HR-B/V test, pathways associated with viral life cycle (6 genes) and viral process (6 genes) were the most common. Four genes were represented in pathways associated with cell killing, biological process involved in symbiotic interaction, defense response to virus, and defense response to symbiont. Three genes were represented in pathways such as type I interferon-mediated signaling pathway, regulation of cell killing, regulation of leukocyte-mediated cytotoxicity, and viral genome replication. Figure 2 displays the 20 pathways in which the classifier genes were represented with the highest statistical significance. Supplementary Table 1 shows the total of 43 pathways in which these genes were represented with p-value less than 0.05.

### Prediction of viral versus bacterial infection using existing blood-based Biomeme Franklin^TM^ models

**Figure 3.**
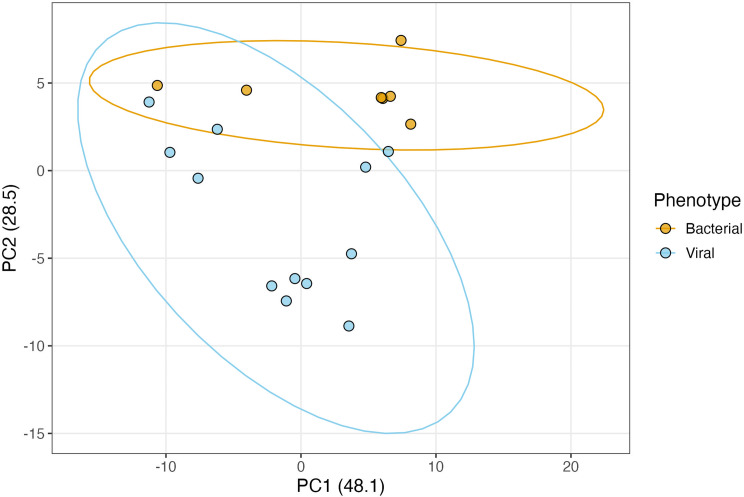
Principal component analysis (PCA) of viral and bacterial infection among patients with lower respiratory tract infection.

We first assessed discrimination of viral and bacterial infection using principal component analysis (PCA) (Figure 3).

**Figure 4.**
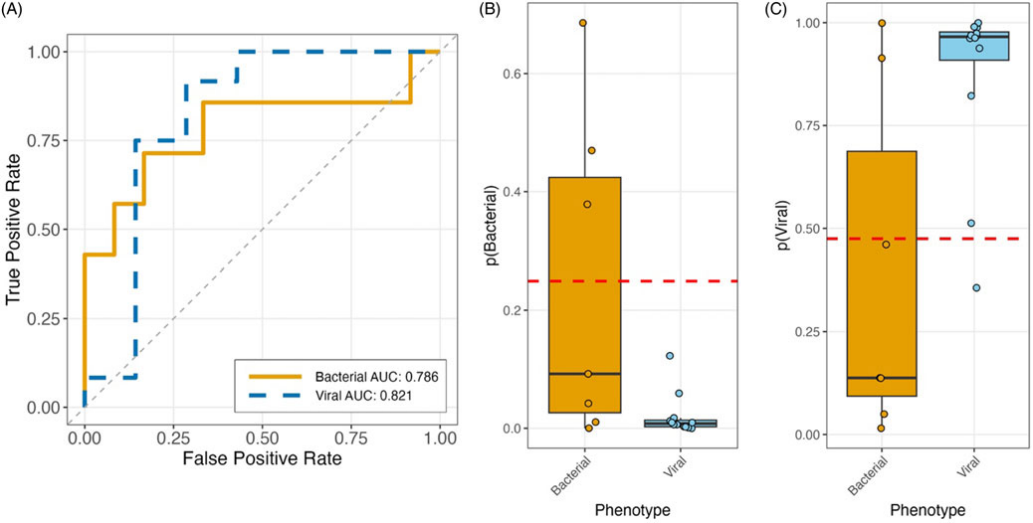
(A) The area under the curves (AUC) and discrimination of viral and bacterial lower respiratory tract infection when using nasopharyngeal swab samples and the existing blood-based Biomeme HR-B/V Franklin^TM^ models. (B) Bacterial model. (C) Viral model. p stands for probability in the figures.

We used the existing blood-based Biomeme HR-B/V Franklin^TM^ models to assess performance at identifying viral versus bacterial infection. When using NP swab samples, the bacterial model AUC was 0.786 (95% CI 0.524–1), with accuracy of 0.79 (95% CI 0.57–0.91), PPA of 0.43 (95% CI 0.16–0.75), and NPA of 1.00 (95% CI 0.76–1) compared to clinical adjudication (Figure 4). The viral model showed similar performance with an AUC of 0.821 (95% CI 0.564–1), accuracy of 0.84 (95% CI 0.62–0.94), PPA of 0.92 (95% CI 0.65–0.99), and NPA of 0.71 (95% CI 0.36–0.92).

### Prediction of viral versus bacterial infection using new model

**Figure 5.**
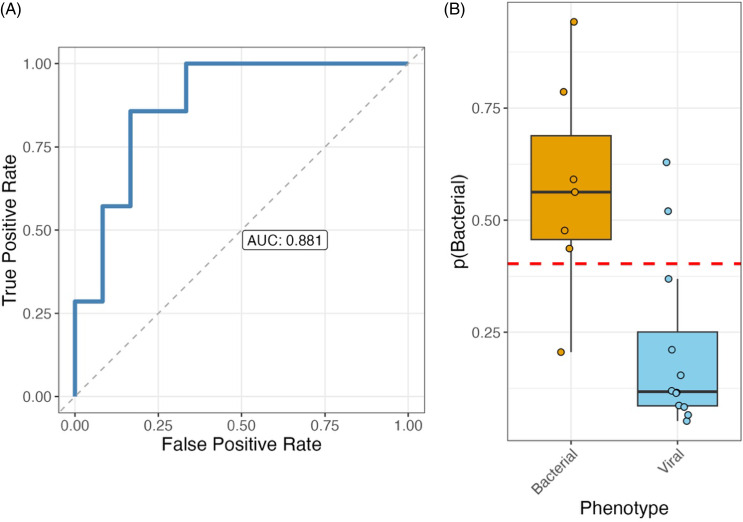
(A) The area under the curve (AUC) and (B) discrimination of viral and bacterial lower respiratory tract infection when using nasopharyngeal samples and a newly derived model. p stands for probability in the figures.

All genes present in the Biomeme HR-B/V test were then used to build a novel predictive model based on NP-derived gene expression data. The AUC of this new model was 0.881 (95% CI 0.726–1). The model had accuracy of 0.84 (95% CI 0.62–0.94), PPA of 0.86 (95% CI 0.49–0.97), and NPA of 0.83 (95% CI 0.55–0.95) for bacterial infection. Figure 5 shows the AUC and the discrimination of viral and bacterial infection, and Supplementary Figure 2 displays the frequency of regression coefficients and the regression coefficient values.

### Comparison to the biomarker CRP

We compared the performance of our newly developed model versus the standard biomarker, CRP (Table 2). Our model showed superior performance to CRP across all metrics, except in the case of PPA, when it displayed equal performance (85.7%). Accuracy of the new model was 77.8% compared to 61.1% for CRP. A test of two proportions confirmed that there were no statistically significant performance differences; this is likely due to the small sample size.

**Table 2.** Performance metrics of the newly developed nasopharyngeal B/V model and C-reactive protein

Group	Accuracy %	PPA %	NPA %	PPV %	NPV %
**Nasopharyngeal** **B/V model**	77.8 (54.8–91.0)	85.7 (48.7–97.4)	72.7 (43.4–90.3)	66.7 (35.4–87.9)	88.9 (56.5–98.0)
**C-reactive protein**	61.1 (38.6–79.7)	85.7 (48.7–97.4)	45.5 (21.3–72.0)	50.0 (25.4–74.6)	83.3 (43.7–97.0)

95% confidence intervals are displayed in parenthesis. Abbreviations: B/V = bacterial/ viral; PPA = positive percent agreement; NPA = negative percent agreement; PPV = positive predictive value; NPV = negative predictive value.

### Validation in external datasets

To establish the generalizability of our results, we validated the Biomeme HR-BV classifier (gene targets) in NP samples using two external gene expression datasets (GSE163151 and GSE188678; Table 3). Few publicly available datasets with NP gene expression data in respiratory infection were found, and these particular datasets were selected because they included patients with both viral and non-viral acute respiratory illness, included RNA sequence data from nasal or NP specimens, and were thought to be most representative of the current dataset of adults with LRTI. GSE163151 included 340 NP samples (258 viral, 82 non-viral) from individuals with suspected respiratory infection. The cohort included 138 patients with COVID-19, 120 patients with other viral infections such as influenza A, influenza B, and rhinovirus, and 82 patients with no virus detected and presumed to be having non-viral respiratory illness. Mean age was 49 ± 20 years in patients with viral infection and 44 ± 16 years in patients who were viral negative. A total of 48% in the viral-positive group were male, compared with 26.5% in the viral-negative group. The majority of patients were ambulatory (70% in viral positive group and 80% in the viral negative group).

**Table 3.** External datasets of patients with viral versus non-viral respiratory illness and RNA sequence data from nasal/ nasopharyngeal samples. The biomeme HR-B/V classifier was validated in these external datasets. 95% confidence intervals (CI) for the area under the curve (AUC) are given in parentheses

	Cohort characteristics	Clinical assignment			
		Viral	Non-viral illness	Total	AUC
GSE163151	Ambulatory and hospitalized patients with COVID-19, other viral respiratory infections (including influenza A and B, rhinovirus, and respiratory syncytial virus), and non-viral respiratory illness.	258	82	340	0.932 (0.901–0.964)
GSE188678	Adults with viral infections (including 90 with COVID-19, 59 with other viral respiratory infections consisting mostly of rhinovirus and influenza), and 169 with no virus detected and presumed to be having non-viral respiratory illness.	149	169	318	0.915 (0.880–0.950)

Abbreviations: AUC = area under the receiver operating characteristic curve.

GSE188678 consisted of adults with acute respiratory illness, with 137 (43.1%) being male and age range consisting of 19–89 years. This cohort included 149 patients with viral infections (including 90 with COVID-19 and 59 with other viral respiratory infections consisting mostly of rhinovirus and influenza), and 169 with no virus detected and presumed to have non-viral respiratory illness.

**Figure 6.**
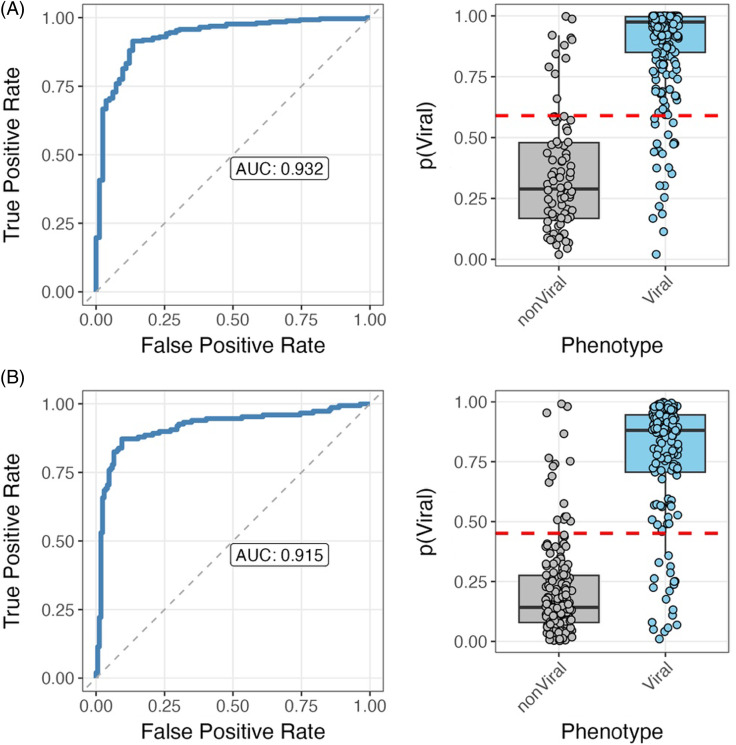
Area under the curve (AUC) (left) and discrimination of viral and non-viral lower respiratory tract infection (right) of the novel NP-derived classifier in two external datasets with nasal or nasopharyngeal RNA sequence data: GSE163151 (A) and GSE188678 (B).

In dataset GSE163151, 23 out of the 24 HR-B/V target genes were found, with HLA-DRB1 missing. Ten of the 23 HR-B/V target genes (SLC29A1, DEFA3, LAPTM4B, DSC2, GCAT, EXOG, FOLR3, BATF, SIRPB1, STAP1) were filtered out of the dataset due to low expression values. Eleven of the 13 remaining genes were found to be expressed at statistically significantly different levels between the viral and non-viral groups. When applying this new RNA-seq NP-derived model, the AUC was 0.932 (95% CI 0.901–0.964) in this dataset (Figure 6a). In dataset GSE188678, all 24 HR-B/V target genes were found. Two of the 24 HR-B/V target genes (DEFA3, FOLR3) were filtered out of the dataset due to low expression values. Eleven of the 22 genes were found to be expressed at statistically significantly different levels between the viral and non-viral groups. The AUC of this RNA-seq NP-derived model was 0.915 (95% CI 0.880–0.950) in this dataset (Figure 6b). Regression coefficient frequency and values are also summarized as bar and box plots in Supplemental Figure 3.

## Discussion

Characterizing the host response to infection is emerging as an important strategy by which viral infection can be distinguished from bacterial infection, and by which infection can be distinguished from colonization. Traditionally, host-based diagnostics have utilized peripheral blood to characterize host response. However, using our prior blood-based classifier, we show that host response in the nasopharynx can also distinguish viral versus bacterial infection, specifically in the lower respiratory tract. Performance was further enhanced by training a classification model on NP-derived gene expression data. Being able to identify viral versus bacterial LRTI using a non-invasively collected sample has important implications for identifying LRTI etiology, which remains unknown in the majority of cases. In addition, using a NP sample affords the possibility of developing an integrated pathogen and host-based diagnostic in the future which identifies infectious etiology using a single, non-invasively collected sample.

We showed that the median expression of certain genes (IFI27, HLA-DRB1, USP18, STAP1, and OAS3) in the Biomeme HR-B/V classifier was higher in viral versus bacterial infection. The pattern of expression for several of these genes is similar to what has been shown previously, and with what would be expected based on known biological function of these genes. For example, IFI27, which is induced by interferon, has been shown to be upregulated in both blood and NP samples in response to viral respiratory infection [32,33]. USP18, STAP1, and OAS3 are involved in anti-viral response and have been shown to be upregulated in blood or lung tissue during viral infection [34–37]. The median expression of some genes (CTBP1, LAPTM4B, CFAP45, DSC2, LAMP1, EXOG, RPS21, and SIRPB1) was higher in bacterial compared to viral infection. While genes present in bacterial pathways are less clearly defined, SIRPB1 has been shown to be involved in the promotion of phagocytosis in macrophages [38]. Pathway analysis showed that the genes in the classifier were represented in pathways such as those associated with viral life cycle, cell killing, and regulation of leukocyte-mediated cytotoxicity.

The use of our existing blood-based Biomeme HR-B/V Franklin^TM^ bacterial model on NP samples showed moderate performance with AUC of 0.786 and accuracy of 0.79 at identifying viral versus bacterial infection. Training on NP-derived gene expression showed improved performance with AUC of 0.881 and accuracy of 0.84. These latter metrics are comparable to those observed in blood with the HR-B/V classifier [10]. Others have previously shown that NP-based classifiers can perform comparably to blood-based classifiers at identifying viral versus non-viral respiratory illness; however, none have directly compared the performance of a classifier on NP versus blood samples [12,39]. From a biological perspective, it is plausible that gene expression changes in the HR-B/V classifier’s targets, many of which are related to immune function, would be similar in the blood versus NP spaces. The high performance of our HR-B/V signature (gene targets) in two external datasets with nasal/ NP samples lends further weight to the biological importance of these genes and to the generalizability of our results. It must be noted that of 32 initial NP samples, 13 (11 viral and 2 bacterial, 41% of total samples) had > 33% of their targets missing and were excluded from subsequent analyses. This level of missingness may be related to variations in sample quality due to collection methods, or may be related to underlying biological differences in gene expression in blood versus the nasopharynx. For example, the expression level of some genes may be too low in NP samples, resulting in the failure to detect them using PCR. Larger studies need to be conducted, and *de novo* classifiers for the NP space need to be explored. The level of missingness may also pose challenges in the future for developing a viable NP-based host response diagnostic that can be used clinically. It is possible that genes selected for such a classifier should be restricted to those with baseline high levels of expression.

The ability of the classifier to identify viral versus bacterial infection in the NP space is promising, as this provides an avenue for an integrated pathogen-host response diagnostic that utilizes a single, non-invasively collected patient sample. No such diagnostics currently exist in clinical care for the diagnosis of respiratory or other infectious syndromes. For respiratory infections, and LRTI in particular, identification of an organism from an upper respiratory sample does not necessarily indicate infection with that organism, thus such a diagnostic could transform current clinical practice. Improved diagnostics may help decrease antibacterial overuse for respiratory viral infections, which has been documented in both inpatient and outpatient settings globally [40–43]. Antibacterial overuse is associated with downstream antimicrobial resistance, which at current rates is estimated to result in 39 million deaths by 2050 [44].

Some limitations must be noted. Our sample size was small. However, this pilot study is an initial proof-of-concept assessment and provides promising results that the HR-B/V assay may work well in NP samples. In addition, the replication of our findings using two external cohorts is a strength and suggests that our results are generalizable. However, our findings need to be further validated with additional internal cohorts as well as multi-site cohorts. The reference standard based on clinical adjudication may have resulted in misclassification. However, we attempted to minimize the chance for misclassification by using a rigorous adjudication system and by only utilizing cases in whom there was a high level of microbiological confirmation. Our results may thus not be applicable to other patients with less definite infection; however, we intend to study the performance of the HR-B/V classifier in the NP samples of patients with indeterminate etiology of infection in future work.

In conclusion, we show that our prior blood-based Biomeme HR-B/V classifier had high performance at identifying viral versus bacterial LRTI, particularly when trained on NP samples. Our findings need to be replicated in larger, multi-center cohorts with diverse etiologies of acute respiratory tract infection.

## Supporting information

10.1017/cts.2025.10191.sm001 Tillekeratne et al. supplementary material Tillekeratne et al. supplementary material

## Supplementary material

The supplementary material for this article can be found at https://doi.org/10.1017/cts.2025.10191.
