# Supplementary material for: Host gene expression in the Nasopharynx can discriminate microbiologically confirmed viral and bacterial lower respiratory tract infection
Source: J Clin Transl Sci. 2025 Oct 29;9(1):e257. doi: 10.1017/cts.2025.10191 (PMC12766521; doi:10.1017/cts.2025.10191)
Supplement: Tillekeratne et al. supplementary material [file S205986612510191Xsup001.docx]

**Supplementary Data**


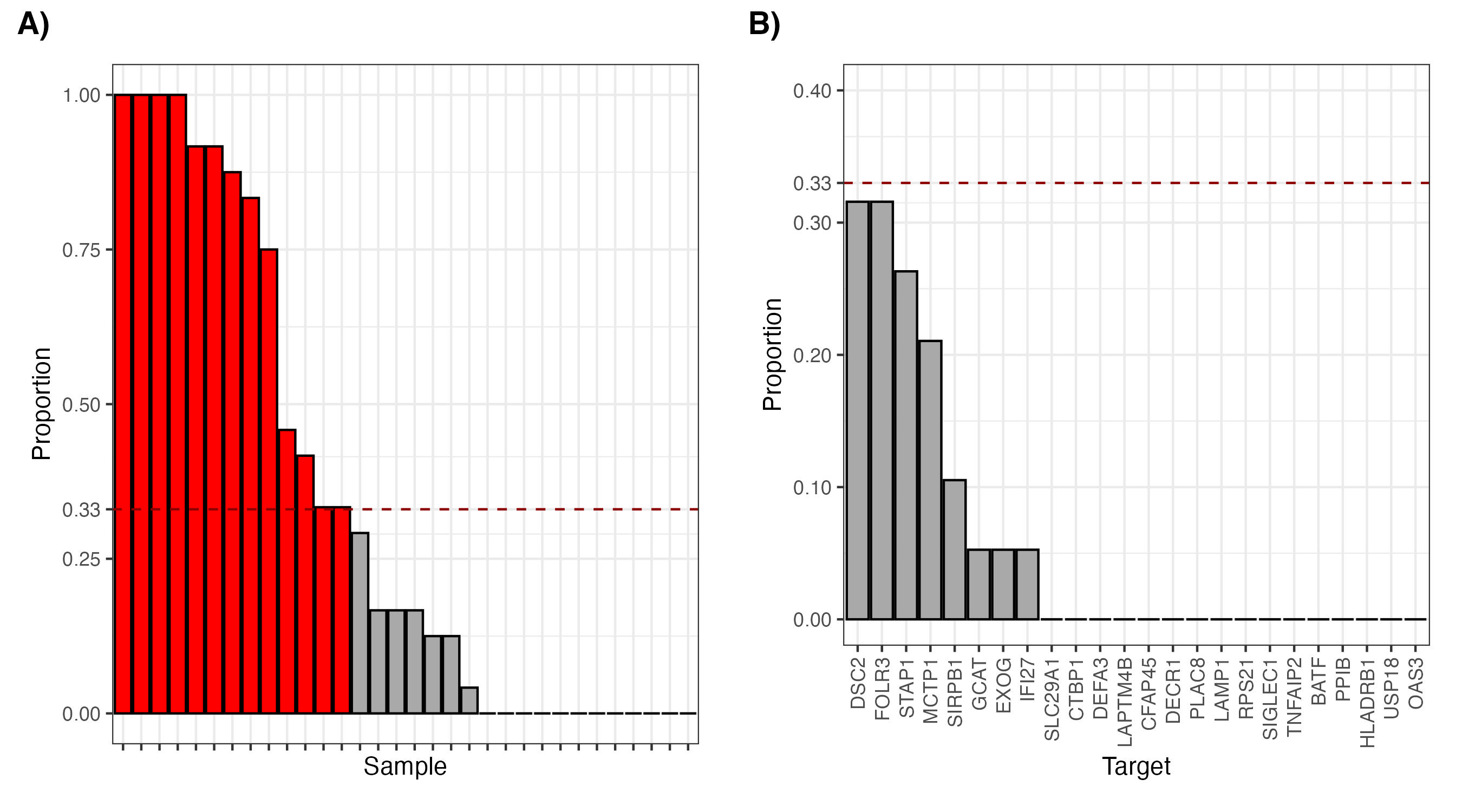


**Supplementary Figure 1**: **A)** Missingness of gene targets from the Biomeme Franklin^TM^ HR-B/V classifier in the nasopharyngeal samples of 32 initial patients. The dotted line shows the 33% missingness threshold at which samples were excluded from this analysis. Missingness was not associated with infection (fisher exact test p-value 0.25). **B)** Proportion of gene targets missing across samples, after removal of low-quality samples, among Sri Lankan patients with viral and bacterial lower respiratory tract infection.


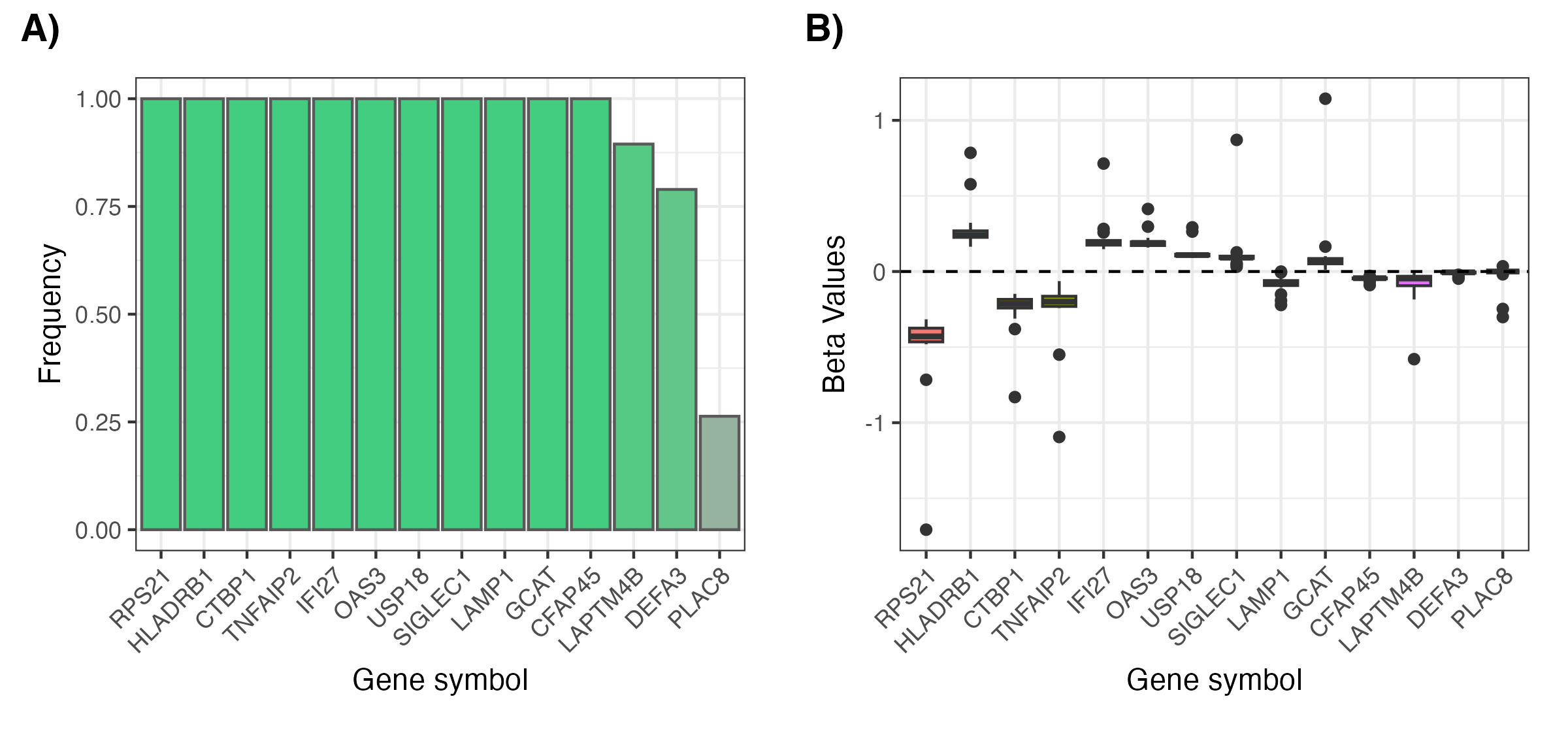


**Supplementary Figure 2:** **A)** Frequency of regression coefficients as they appear in the leave-one-out cross-validation (LOOCV) nested loop and **B)** the regression coefficient values for viral versus bacterial infection when using nasopharyngeal samples and a newly derived model.


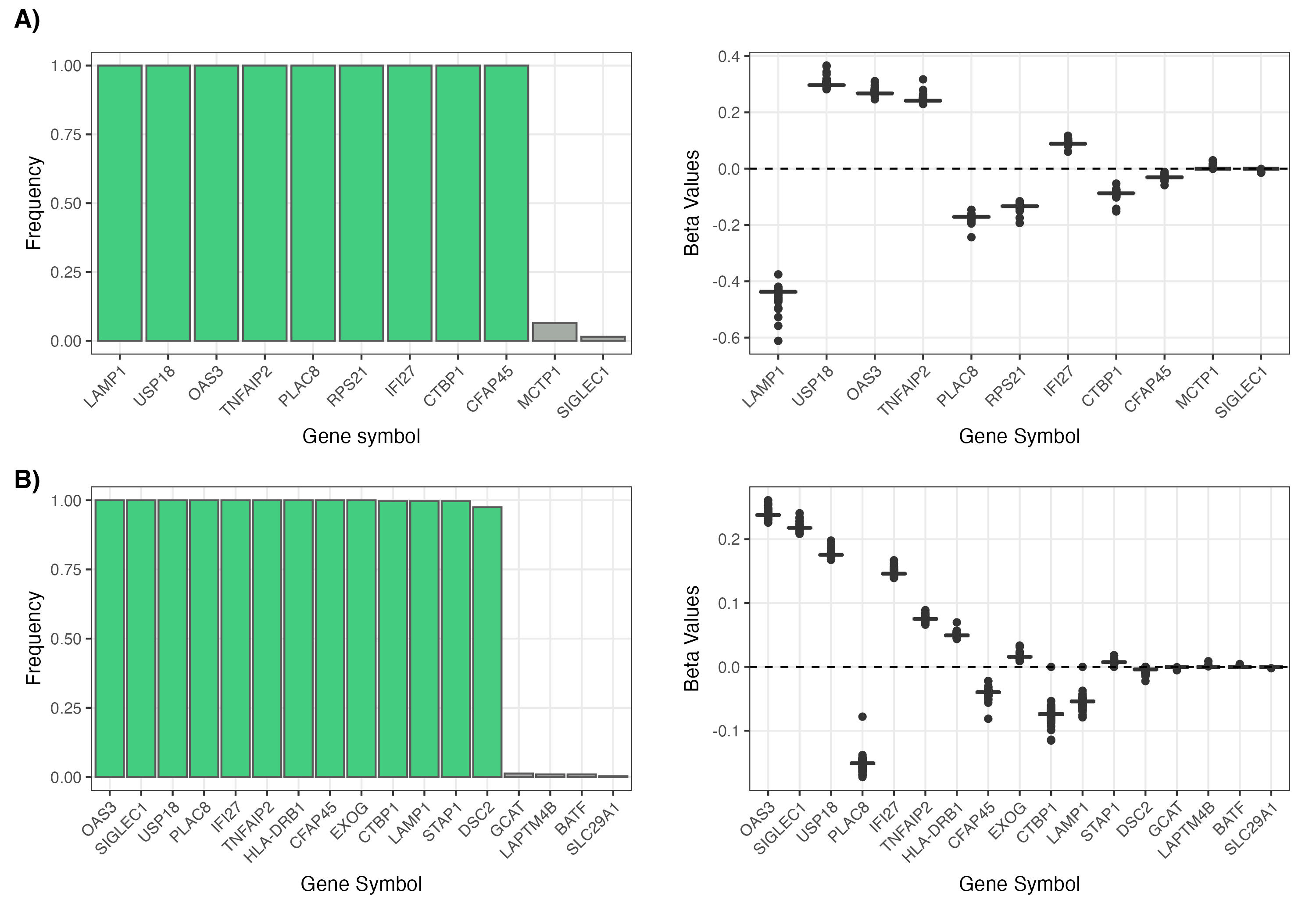


**Supplementary Figure 3:** External validation of the Biomeme Franklin^TM^ HR-B/V classifier when assessing datasets of patients with respiratory infection and nasal or nasopharyngeal RNA sequence data. **A)** Frequency of regression coefficients as they appear in the leave-one-out cross-validation (LOOCV) nested loop (left) and the regression coefficient values for viral versus bacterial infection (right) for GSE163151. **B)** Frequency of regression coefficients as they appear in the LOOCV nested loop (left) and the regression coefficient values for viral versus bacterial infection (right) for GSE188678.

**Supplementary Table 1:**

All pathways in which the 22 genes represented in the Biomeme Franklin^TM^ HR-B/V classifier were found at a statistically significant level compared to other pathways. All pathways with p-value less than 0.05 are displayed here. Gene ratio is defined as the number of targets found within a specific pathway, over all the targets in the classifier. BgRatio is defined as the number of genes that annotate to a pathway, over the entire background distribution (universe).

| **ID** | **Description of pathway** | **Gene ratio** | **BgRatio** | **p-value** | **Adjusted p-value** | **Genes** |
| --- | --- | --- | --- | --- | --- | --- |
| GO:0019058 | viral life cycle | 6/22 | 318/18280 | 1.5597E-06 | 0.001 | CTBP1/LAMP1/SIGLEC1/IFI27/HLA-DRB1/OAS3 |
| GO:0016032 | viral process | 6/22 | 426/18280 | 8.4053E-06 | 0.003 | CTBP1/LAMP1/SIGLEC1/IFI27/HLA-DRB1/OAS3 |
| GO:0001912 | positive regulation of leukocyte mediated cytotoxicity | 3/22 | 67/18280 | 0.000068948 | 0.012 | LAMP1/HLA-DRB1/STAP1 |
| GO:0031343 | positive regulation of cell killing | 3/22 | 74/18280 | 0.000092791 | 0.012 | LAMP1/HLA-DRB1/STAP1 |
| GO:0001906 | cell killing | 4/22 | 216/18280 | 0.00011735 | 0.012 | DEFA3/LAMP1/HLA-DRB1/STAP1 |
| GO:0060337 | type I interferon-mediated signaling pathway | 3/22 | 81/18280 | 0.00012147 | 0.012 | IFI27/USP18/OAS3 |
| GO:0071357 | cellular response to type I interferon | 3/22 | 82/18280 | 0.00012598 | 0.012 | IFI27/USP18/OAS3 |
| GO:0001960 | negative regulation of cytokine-mediated signaling pathway | 3/22 | 86/18280 | 0.00014513 | 0.012 | USP18/STAP1/OAS3 |
| GO:0034340 | response to type I interferon | 3/22 | 88/18280 | 0.00015538 | 0.012 | IFI27/USP18/OAS3 |
| GO:0060761 | negative regulation of response to cytokine stimulus | 3/22 | 91/18280 | 0.00017161 | 0.012 | USP18/STAP1/OAS3 |
| GO:0001910 | regulation of leukocyte mediated cytotoxicity | 3/22 | 94/18280 | 0.00018891 | 0.012 | LAMP1/HLA-DRB1/STAP1 |
| GO:0140888 | interferon-mediated signaling pathway | 3/22 | 106/18280 | 0.00026935 | 0.014 | IFI27/USP18/OAS3 |
| GO:0060339 | negative regulation of type I interferon-mediated signaling pathway | 2/22 | 21/18280 | 0.00028636 | 0.014 | USP18/OAS3 |
| GO:0031341 | regulation of cell killing | 3/22 | 109/18280 | 0.00029242 | 0.014 | LAMP1/HLA-DRB1/STAP1 |
| GO:0019079 | viral genome replication | 3/22 | 130/18280 | 0.00049024 | 0.022 | CTBP1/IFI27/OAS3 |
| GO:0044403 | biological process involved in symbiotic interaction | 4/22 | 318/18280 | 0.00051315 | 0.022 | LAMP1/SIGLEC1/IFI27/HLA-DRB1 |
| GO:0001909 | leukocyte mediated cytotoxicity | 3/22 | 136/18280 | 0.00055926 | 0.022 | LAMP1/HLA-DRB1/STAP1 |
| GO:0051607 | defense response to virus | 4/22 | 331/18280 | 0.00059664 | 0.022 | DEFA3/IFI27/USP18/OAS3 |
| GO:0140546 | defense response to symbiont | 4/22 | 332/18280 | 0.00060344 | 0.022 | DEFA3/IFI27/USP18/OAS3 |
| GO:0002705 | positive regulation of leukocyte mediated immunity | 3/22 | 148/18280 | 0.00071534 | 0.025 | LAMP1/HLA-DRB1/STAP1 |
| GO:0001818 | negative regulation of cytokine production | 4/22 | 358/18280 | 0.0008003 | 0.026 | LAPTM4B/SIGLEC1/HLA-DRB1/OAS3 |
| GO:0046718 | viral entry into host cell | 3/22 | 155/18280 | 0.000818 | 0.026 | LAMP1/SIGLEC1/HLA-DRB1 |
| GO:0002696 | positive regulation of leukocyte activation | 4/22 | 372/18280 | 0.00092332 | 0.027 | LAMP1/HLA-DRB1/SIRPB1/STAP1 |
| GO:0044409 | entry into host | 3/22 | 162/18280 | 0.00092962 | 0.027 | LAMP1/SIGLEC1/HLA-DRB1 |
| GO:0002697 | regulation of immune effector process | 4/22 | 383/18280 | 0.001 | 0.027 | LAMP1/BATF/HLA-DRB1/STAP1 |
| GO:0001959 | regulation of cytokine-mediated signaling pathway | 3/22 | 168/18280 | 0.001 | 0.027 | USP18/STAP1/OAS3 |
| GO:0050867 | positive regulation of cell activation | 4/22 | 387/18280 | 0.001 | 0.027 | LAMP1/HLA-DRB1/SIRPB1/STAP1 |
| GO:0045622 | regulation of T-helper cell differentiation | 2/22 | 42/18280 | 0.001 | 0.028 | BATF/HLA-DRB1 |
| GO:0060338 | regulation of type I interferon-mediated signaling pathway | 2/22 | 43/18280 | 0.001 | 0.029 | USP18/OAS3 |
| GO:0060759 | regulation of response to cytokine stimulus | 3/22 | 180/18280 | 0.001 | 0.029 | USP18/STAP1/OAS3 |
| GO:0044000 | movement in host | 3/22 | 186/18280 | 0.001 | 0.031 | LAMP1/SIGLEC1/HLA-DRB1 |
| GO:0009615 | response to virus | 4/22 | 429/18280 | 0.002 | 0.034 | DEFA3/IFI27/USP18/OAS3 |
| GO:0050777 | negative regulation of immune response | 3/22 | 200/18280 | 0.002 | 0.036 | HLA-DRB1/USP18/OAS3 |
| GO:0002443 | leukocyte mediated immunity | 4/22 | 448/18280 | 0.002 | 0.036 | LAMP1/BATF/HLA-DRB1/STAP1 |
| GO:0060632 | regulation of microtubule-based movement | 2/22 | 53/18280 | 0.002 | 0.036 | CFAP45/LAMP1 |
| GO:0043370 | regulation of CD4-positive, alpha-beta T cell differentiation | 2/22 | 54/18280 | 0.002 | 0.036 | BATF/HLA-DRB1 |
| GO:0051701 | biological process involved in interaction with host | 3/22 | 209/18280 | 0.002 | 0.036 | LAMP1/SIGLEC1/HLA-DRB1 |
| GO:0002285 | lymphocyte activation involved in immune response | 3/22 | 212/18280 | 0.002 | 0.036 | LAMP1/BATF/HLA-DRB1 |
| GO:0032102 | negative regulation of response to external stimulus | 4/22 | 462/18280 | 0.002 | 0.036 | HLA-DRB1/USP18/STAP1/OAS3 |
| GO:0051249 | regulation of lymphocyte activation | 4/22 | 498/18280 | 0.003 | 0.046 | LAMP1/BATF/HLA-DRB1/SIRPB1 |
| GO:0002683 | negative regulation of immune system process | 4/22 | 500/18280 | 0.003 | 0.046 | HLA-DRB1/USP18/STAP1/OAS3 |
| GO:0002703 | regulation of leukocyte mediated immunity | 3/22 | 245/18280 | 0.003 | 0.049 | LAMP1/HLA-DRB1/STAP1 |
| GO:0050766 | positive regulation of phagocytosis | 2/22 | 69/18280 | 0.003 | 0.049 | SIRPB1/STAP1 |
